# Pathogenic Mouse Hepatitis Virus or Poly(I:C) Induce IL-33 in Hepatocytes in Murine Models of Hepatitis

**DOI:** 10.1371/journal.pone.0074278

**Published:** 2013-09-13

**Authors:** Muhammad Imran Arshad, Solène Patrat-Delon, Claire Piquet-Pellorce, Annie L’Helgoualc’h, Michel Rauch, Valentine Genet, Catherine Lucas-Clerc, Christian Bleau, Lucie Lamontagne, Michel Samson

**Affiliations:** 1 Institut de Recherche Santé Environnement et Travail (IRSET) - U.1085, Institut National de la Santé et de la Recherche Médicale (Inserm), Rennes, Bretagne, France; 2 Université de Rennes 1, Rennes, Bretagne, France; 3 Structure Fédérative BioSit UMS 3480 CNRS-US18 Inserm, Rennes, Bretagne, France; 4 Service de Biochimie CHU Rennes, Université de Rennes 1, Rennes, Bretagne, France; 5 Département des Sciences Biologiques, Université du Québec à Montréal, Montréal, Québec, Canada; 6 Institute of Microbiology, University of Agriculture, Faisalabad, Pakistan; CNRS, France

## Abstract

The IL-33/ST2 axis is known to be involved in liver pathologies. Although, the IL-33 levels increased in sera of viral hepatitis patients in human, the cellular sources of IL-33 in viral hepatitis remained obscure. Therefore, we aimed to investigate the expression of IL-33 in murine fulminant hepatitis induced by a Toll like receptor (TLR3) viral mimetic, poly(I:C) or by pathogenic mouse hepatitis virus (L2-MHV3). The administration of poly(I:C) plus D-galactosamine (D-GalN) in mice led to acute liver injury associated with the induction of IL-33 expression in liver sinusoidal endothelial cells (LSEC) and vascular endothelial cells (VEC), while the administration of poly(I:C) alone led to hepatocyte specific IL-33 expression in addition to vascular IL-33 expression. The hepatocyte-specific IL-33 expression was down-regulated in NK-depleted poly(I:C) treated mice suggesting a partial regulation of IL-33 by NK cells. The CD1d KO (NKT deficient) mice showed hepatoprotection against poly(I:C)-induced hepatitis in association with increased number of IL-33 expressing hepatocytes in CD1d KO mice than WT controls. These results suggest that hepatocyte-specific IL-33 expression in poly(I:C) induced liver injury was partially dependent of NK cells and with limited role of NKT cells. In parallel, the L2-MHV3 infection in mice induced fulminant hepatitis associated with up-regulated IL-33 expression as well as pro-inflammatory cytokine microenvironment in liver. The LSEC and VEC expressed inducible expression of IL-33 following L2-MHV3 infection but the hepatocyte-specific IL-33 expression was only evident between 24 to 32h of post infection. In conclusion, the alarmin cytokine IL-33 was over-expressed during fulminant hepatitis in mice with LSEC, VEC and hepatocytes as potential sources of IL-33.

## Introduction

Interleukin-33 (IL-33), a member of IL-1 family also called as IL-1F11, is known to drive immune responses by interaction with its specific receptors ST2 and IL-RAcP [1,2]. The IL-33/ST2 axis is crucially involved in diverse inflammatory and immune mediated pathologies [3,4]. However, limited data is available about the association of IL-33 and ST2 expression in viral diseases. IL-33 is over-expressed in influenza virus lung infection in mice [5,6] and IL-33 produced by necrotic cells drives protective antiviral CD8^+^ T cell responses in lymphocytic choriomeningitis virus (LCMV) infection in mice [7]. Further, elevated levels of soluble ST2 (sST2) in sera of dengue virus infected patients [8] and HIV infected patients [9] were observed indicating sST2 as potential marker of viral infections.

In liver, IL-33/ST2 axis is involved in various viral and immune cell mediated pathologies [10-13]. We initially observed up-regulated expression of IL-33 and ST2 in chronic hepatitis B and C virus (HBV and HCV) infection in human and in CCl_4_-induced liver fibrosis in mice [14]. The increased level of serum IL-33 and sST2 was observed in acute and chronic hepatic failure in human [15]. Furthermore, elevated IL-33 serum level was also associated with liver damage in patients of chronic hepatitis C virus (HCV) [16] and hepatitis B virus (HBV) [17] infections, representing IL-33 as a possible indicator of viral hepatitis.

Despite the fact that IL-33 is proposed to be released as an alarmin in acute inflammatory pathologies [3], the expression and cellular sources of IL-33 during viral fulminant hepatitis in a relevant animal model has not been explored. The polyinosine-polycytidylic acid (Poly(I:C), a synthetic analog of double stranded RNA (dsRNA), induces a moderate acute hepatic injury and mimics a model of viral hepatitis [18,19]. Poly(I:C) activated principally the intrahepatic macrophages (Kuppfer cells) and NK cells via TLR3 [20] leading to increase of inflammatory cytokines such as TNF-α, IFN-γ, IL-6, IL-12 and IFN-β [18,19,21,22]. A pretreatment with D-galactosamine (D-GalN) in Poly(I:C) injected-mice aggravated the acute hepatic injury which become lethal [19]. A natural animal model of viral hepatitis, the mouse hepatitis viruses (MHV), single-strand, positive-sense RNA viruses belonging to *Coronaviridae* family, induced acute and/or chronic hepatitis in mice mimicking human HBV infection and serve as a good tool to study immune dysfunction and cytokines associated with viral acute hepatitis [23,24]. The most hepatotropic serotype of MHV, the mouse hepatitis virus type 3 (MHV3), induced severe fulminant hepatitis in mice and their death within 3-5 days post-infection [25]. In liver, Kupffer cells, NK cells, hepatocytes, sinusoidal endothelial and vascular endothelial cells are the main target cells for MHV3 replication [26,27]. The histopathological lesions in liver were correlated with the levels of inflammatory cytokines [28]. High levels of IL-6 and TNF-α produced in livers from infected C57BL/6 mice were modulated by TLR receptor. We have previously demonstrated that intrahepatic NK cells decreased after a transient increase in liver from pathogenic L2-MHV3-infected mice due to virus-induced NK depletion [27].

In the present study, we aimed to investigate the expression and cellular sources of IL-33 in a Poly(I:C)- and L2-MHV3-induced fulminant hepatitis in mice. We found increased expression of IL-33 in liver following Poly(I:C) and L2-MHV3 induced acute hepatitis in mice. The liver sinusoidal endothelial cells, vascular endothelial cells and hepatocytes represent potential sources of IL-33 in Poly(I:C) and murine L2-MHV3 induced fulminant hepatitis. The hepatocyte-specific IL-33 expression in Poly(I:C) induced liver injury was partially dependent of NK cells but not of NKT cells.

## Materials and Methods

### Animals

Wild-type (WT) C57BL/6 mice were purchased from Charles River Laboratories (St-Constant, QC, Canada) or from Janvier (Le Genest-sur-isle, France). The animals, certified as MHVs-free by the manufacturer, were housed under HEPA-filtered air (Forma Scientific, Marietta, OH). The study was conducted in compliance with the regulations of the Comité institutionnel de la Protection des Animaux of the Université du Québec à Montreal (UQAM agreement of L. Lamontagne, No. CIPA= 541), and French laws and the institution’s guidelines for animal welfare (agreement of M. Samson #3596). The protocol was approved by the Committee on the Ethics of Animal Experiments of the French government (agreement of M. Samson #3596). All efforts were made to minimize suffering"

### In vivo treatment protocol

The C57BL/6 (Janvier, France) or CD1d KO mice (a gift of Maria Leite-de-Moraes, Paris) were intravenously injected with 30 µg/mouse of Poly(I:C) (Invivogen) alone or with D-galactosamine (D-GalN) (SIGMA-G0264) pretreatment at a dose of 15 mg/mouse (i.p). The control mice received similar volume of vehicle in each treatment group. For NK cells depletion experiment, 35 µl of anti-asialo GM1 (anti-ASGM1) polyclonal antibody (Cerdalane, CL8955) was injected intraperitoneally (i.p) 48 h before D-GalN, Poly(I:C) or combination of both D-GalN Poly(I:C) injections, the control mice received or an equivalent amount of naive rabbit serum. The NK depletion in liver was confirmed by flow cytometry in isolated liver immune cells as described earlier [29].

For MHV3 infection in mice, the C57BL/6 mice (Charles River Laboratories, Canada) were infected by the i.p route with 10^3^ 50% tissue culture infective dose (TCID(50)) of pathogenic L2-MHV3 strain as previously described [25]. Mock-infected or uninfected control mice received a similar volume of RPMI-1640 (Gibco Laboratories, Grand Island, NY). After 16, 24, 28, 39, 48 and 72 h of infection, the mice were anaesthetized by i.p injection using ketamine hydrochloride (200 mg/kg; Vetrepharm Canada Inc., Belleville, ON, Canada) and xylazine (10 mg/kg; Bayer Inc., Toronto, ON, Canada) and euthanazied by CO_2_ inhalation before liver and blood sampling.

### Histopathological, biochemical and immunohistochemical analyses

The histopathological (Hematoxylin And Eosin (H&E) staining) and levels of liver transaminase (ALT/AST) in serum were performed as described earlier [10]. Immunolocalisation of IL-33 was performed by immunohistochemical staining using primary antibody goat IgG anti-mouse-IL-33 (R&D Systems) and secondary HRP-conjugated rabbit anti-goat antibody (Dako, USA) followed by hematoxylin counterstaining in Ventana machine (Ventana Medical Systems, Inc., USA). The counting of IL-33 positive hepatocytes was carried in at least 20 different microscopic fields corresponding to 2.67 mm^2^ surface area by using image analysis software (Compix, Inc. HAMAMATSU company, Japan) as previously described [13].

### RNA isolation and RT-qPCR

The protocol and conditions for RNA extraction, RT-PCR and qPCR were similar as reported earlier by our laboratory using specific primers for 18S, IL-33, IL-6, IL-1β, IFN-β, IFNγ, TNFα and CXCL1 [10,13]. For the quantification of viral nucleocapsid, the following primer set was used: 5’-TGGAAGGTCTGCACCTGCTA-3’ (forward), 5’-TTTGGCCCACGGGATTG-3’ (reverse). The relative gene expression was normalized against 18S gene expression. The control mice in each treatment group served as a reference for mRNA expression (control mRNA level was arbitrarily taken as 1).

### Statistical analysis

The results are representative of three independent experiments and expressed as means±SEM. Mann-Whitney *U* test was used for comparison of control group parameters with treatment group and multiple group analysis was evaluated by one-way ANOVA with post Mann-Whitney *U* test using GraphPad Prism5 software. For all statistical analyses, p-values <0.05 were considered significant.

## Results

### Poly(I:C) administration induced acute liver injury in mice with expression of IL-33 in liver

**Figure 1:**
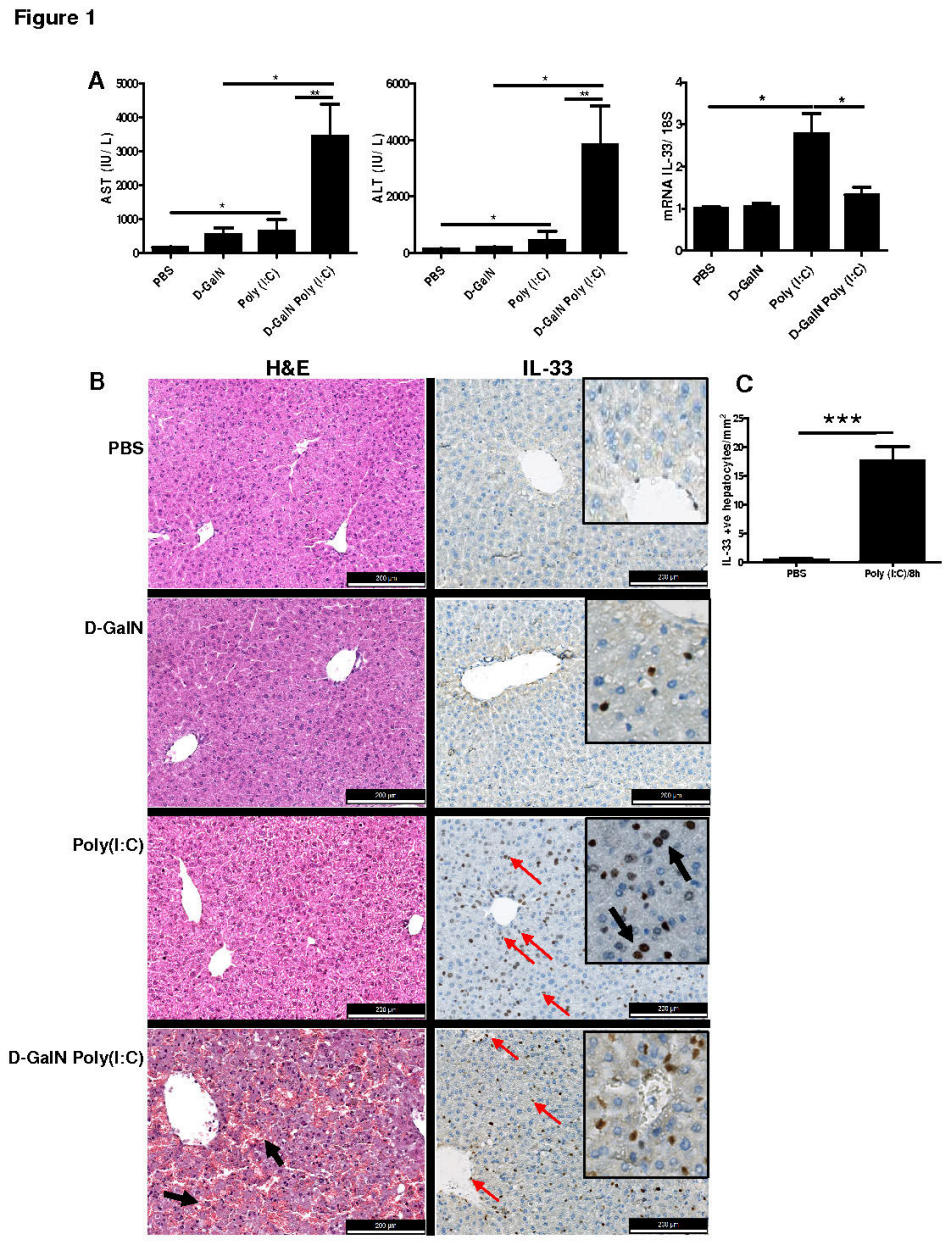
Liver injury and IL-33 expression in D-GalN, Poly(I:C), D-GalN Poly(I:C) treated mice. (**A**) Levels of serum AST/ALT (IU/L) and relative fold change in mRNA expression of IL-33 in WT mice treated with Poly(I:C) (30 µg/mouse i.v.) and/or D-GalN Poly(I:C) at 8h of post injection. (**B**) Sections of mice liver following PBS, D-GalN, Poly(I:C) and D-GalN Poly(I:C) treatment were stained with H&E for histopathology (arrows indicating hemorrhagic lesions in liver) and for immunolocalisation of IL-33 by using primary antibody goat IgG anti-mouse-IL-33 and secondary HRP-conjugated rabbit anti-goat antibody with hematoxylin counterstaining (black arrows and red arrows indicating IL-33 positive hepatocytes and vascular/sinusoidal endothelial cells, respectively). Scale bar was 200 µm. (**C**) Comparison of number of IL-33 expressing hepatocytes in PBS and Poly(I:C) treated mice at 8h.

While the cellular source of IL-33 in viral liver pathology is poorly known in a mouse model, we first aimed to investigate the expression and cellular sources of IL-33 in a Poly(I:C)-induced acute hepatitis. The administration of Poly(I:C) induced moderate liver injury compared to PBS treated mice at 8h, as evident from serum AST/ALT levels (Figure 1A). However, the pre-sensitization of mice with D-galactosamine (D-GalN) led to Poly(I:C)-induced severe liver injury in mice with elevated serum AST/ALT levels at 8h in comparison to D-GalN alone treatment (Figure 1A). The mice treated with combination of D-GalN Poly(I:C) died earlier within 16h compared to D-GalN or Poly(I:C) alone treated mice (Figure S1), therefore, we used 8h time point in this study. The mRNA expression of IL-33 in liver was not significantly increased in Poly(I:C) or D-GalN Poly(I:C) treated mice in comparison with PBS control mice (Figure 1A).

The histology of liver tissues revealed increased hemorrhagic lesions in liver after D-GalN Poly(I:C) treatment but less or no marked liver injury in Poly(I:C) or vehicle control mice (Figure 1B). The mRNA expression of IL-33 was significantly increased (2-3 fold) in Poly(I:C) treated mice compared to control mice, however, IL-33 expression was downregulated in D-GalN Poly(I:C) treated mice in comparison with Poly(I:C) alone (Figure 1A). Regarding cellular sources of IL-33, the liver sinusoidal endothelial cells and vascular endothelial cells expressed IL-33 constitutively in PBS control mice livers and induced expression in these cells was observed following Poly(I:C) and D-GalN Poly(I:C) treatment (Figure 1B). Interestingly, the nuclear expression of IL-33 was found in hepatocytes of Poly(I:C) treated mice (arrows in insert indicate IL-33 positive hepatocytes) but not in D-GalN Poly(I:C) induced liver injury (Figure 1B). The number of IL-33 expressing hepatocytes were clearly and significantly increased in Poly(I:C) induced acute liver injury compared to control mice (Figure 1C). These results suggest that regulation of IL-33 in hepatocytes is associated with Poly(I:C) induced TLR3 stimulation in liver.

### Poly(I:C)-induced hepatitis up-regulated pro-inflammatory cytokine expression in liver

**Figure 2:**
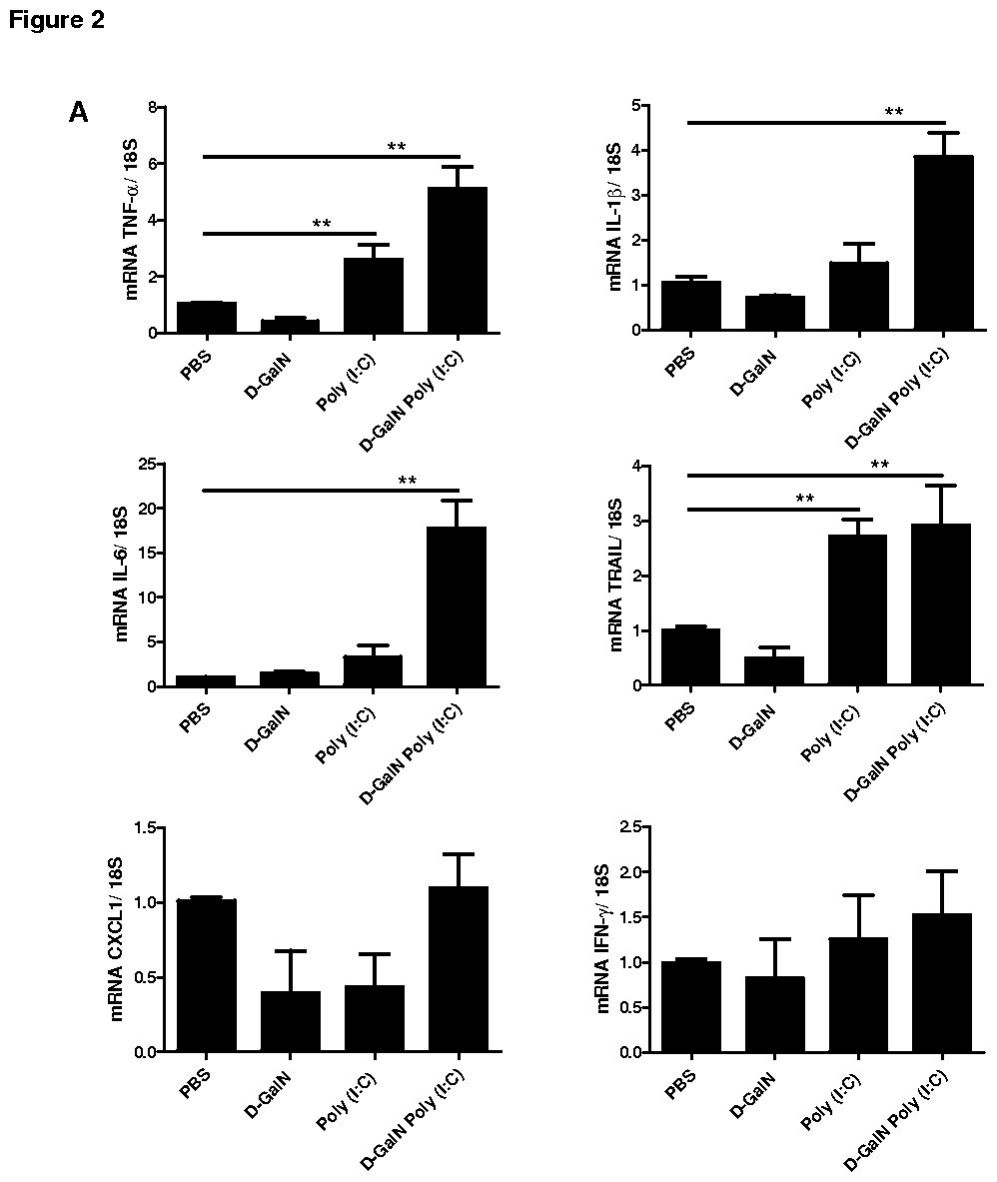
Cytokine expression of TNF-α, IL-1β, IL-6, TRAIL, CXCL1, and IFN-γ in D-GalN, Poly(I:C), D-GalN Poly(I:C) induced hepatitis mice. Relative fold change in mRNA expression of TNF-α, IL-1β, IL-6, TRAIL, CXCL1, and IFN-γ in livers of Poly(I:C) (30 µg/mouse i.v.) and/or D-GalN Poly(I:C) treated mice at 8h of post injection. The PBS-treated mice served as a reference for mRNA expression.

The inflammatory cytokines play an important role in development of fulminant hepatitis. Therefore, we investigated the pro-inflammatory cytokine expression in Poly(I:C) and D-GalN Poly(I:C) induced acute hepatitis. The transcript level of TNF-α, TRAIL, IL-1β and IL-6 was significantly up-regulated following D-GalN Poly(I:C) induced acute hepatitis (8h) compared to D-GalN or PBS control mice (Figure 2). However, the mRNA expression of IFN-γ and CXCL1/KC was not varied between Poly(I:C), D-GalN Poly(I:C) or D-GalN/PBS treated mice (Figure 2).

### NK cells pre-depletion led to severe D-GalN Poly(I:C) induced acute liver injury, increased pro-inflammatory cytokines and down-regulated IL-33 expression in hepatocytes

**Figure 3:**
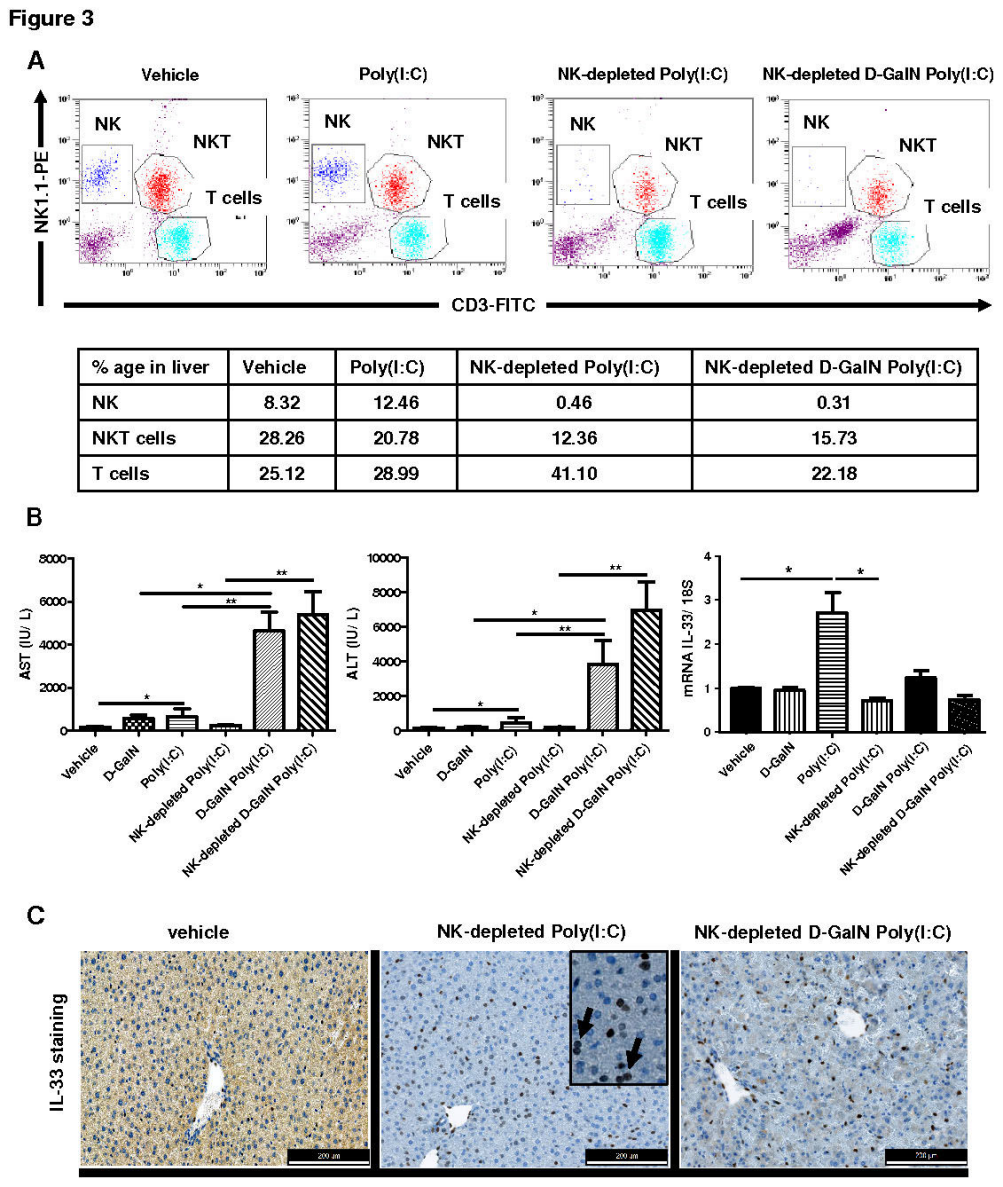
Liver injury and cytokine expression of TNF-α, IL-1β, IL-6, TRAIL, CXCL1, IFN-γ and IL-33 in non NK depleted and NK depleted D-GalN, Poly(I:C), D-GalN Poly(I:C) treated mice. (**A**) Pre-depletion (48h before) of NK cells by anti-ASGM1 antibody in mice and confirmation of NK cells percentage by flow cytometry analysis (CD3-FITC and NK1.1-PE markers) in Vehicle (control), Poly(I:C)-, NK-depleted poly(I-C)-, and NK-depleted D-GalN Poly(I:C)-treated mice (**B**) Levels of serum AST/ALT (IU/L) and relative fold change in mRNA expression of IL-33 in NK depleted or not with Poly(I:C) (30 µg/mouse i.v.) or D-GalN Poly(I:C)-treated mice at 8h of post injection. (**C**) Immunostaining of IL-33 in livers of NK depleted or not with Poly(I:C) (30 µg/mouse i.v.) or D-GalN Poly(I:C) treated mice at 8h of post injection. (**D**) Comparison of number of IL-33 expressing hepatocytes in PBS or Vehicle, Poly(I:C)-treated and NK-depleted Poly(I:C)-treated mice. (**E**) Relative fold change in mRNA expression of TNF-α, IL-1β, IL-6, TRAIL, CXCL1, and IFN-γ in livers of NK depleted or not with Poly(I:C) (30 µg/mouse i.v.) and/or D-GalN Poly(I:C) treated mice (C57Bl/6) at 8h of post injection.

NK cells have shown to be crucially important in Poly(I:C) induced liver injury [22]. Here, we studied the effect of depletion of NK cells by anti-ASGM1 antibody on D-GalN Poly(I:C) induced acute hepatitis and expression of IL-33. As expected, the anti-ASGM1 pre-treatment efficiently depleted NK cells in liver of mice compared to vehicle control or Poly(I:C) treated mice (Figure 3A). However, NK cells depletion led to enhanced liver injury (as evaluated by serum AST/ALT) in D-GalN Poly(I:C) treated mice than Poly(I:C) alone or vehicle control mice (Figure 3B). A milder increase in serum transaminases was evident between D-GalN Poly(I:C) and NK-depleted D-GalN Poly(I:C) treated mice (Figure 3B).

The mRNA expression of IL-33 was not varied between control (non NK depleted) and NK- depleted Poly(I:C) or D-GalN Poly(I:C) treated mice although the expression of IL-33 was diminished in NK-depleted D-GalN Poly(I:C) mice (Figure 3B). The liver sinusoidal endothelial cells (LSEC) and vascular endothelial cells (VEC) expressed IL-33 in NK depleted Poly(I:C) or D-GalN Poly(I:C) treated mice livers and hepatocyte-specific IL-33 expression was evident only after Poly(I:C) treatment (Figure 3C) (arrows indicate IL-33 positive hepatocytes). The NK depletion led to decrease in number of IL-33 expressing hepatocytes in Poly(I:C) treated mice compared to non NK depleted mice (Figure 3D) suggesting a partial NK cells dependent regulation of IL-33 in hepatocytes.

The pro-inflammatory cytokine expression of TNF-α, IL-1β and IL-6 was up-regulated in Poly(I:C) or D-GalN Poly(I:C) treated mice when compared with control mice (p <0.01) (Figure 3E). NK cell depletion did not decrease the expression level of the inflammatory cytokines (Figure 3E). The mRNA expression of IFN-γ was not significantly varied among the treated and control groups of mice except in NK-depleted D-GalN Poly(I:C) treated mice (Figure 3E). The expression of TRAIL was significantly increased in both NK depleted and non depleted mice following Poly(I:C) or D-GalN Poly(I:C) administration when compared with control mice (p <0.01)) but significantly decrease in NK depleted mice after D-GalN Poly(I:C) treatment (p <0.05) when compared with D-GalNPolyIC treated mice (Figure 3E). The expression of CXCL1 only increased in NK depleted D-GalN Poly(I:C) treated mice when compared to control mice (p <0.05) (Figure 3E) that may correlate with development of inflammatory microenvironment in liver and neutrophil migration during liver injury.

### NKT cells deficiency protected mice against Poly(I:C)-induced liver injury but up-regulated hepatocyte-specific IL-33 expression

**Figure 4:**
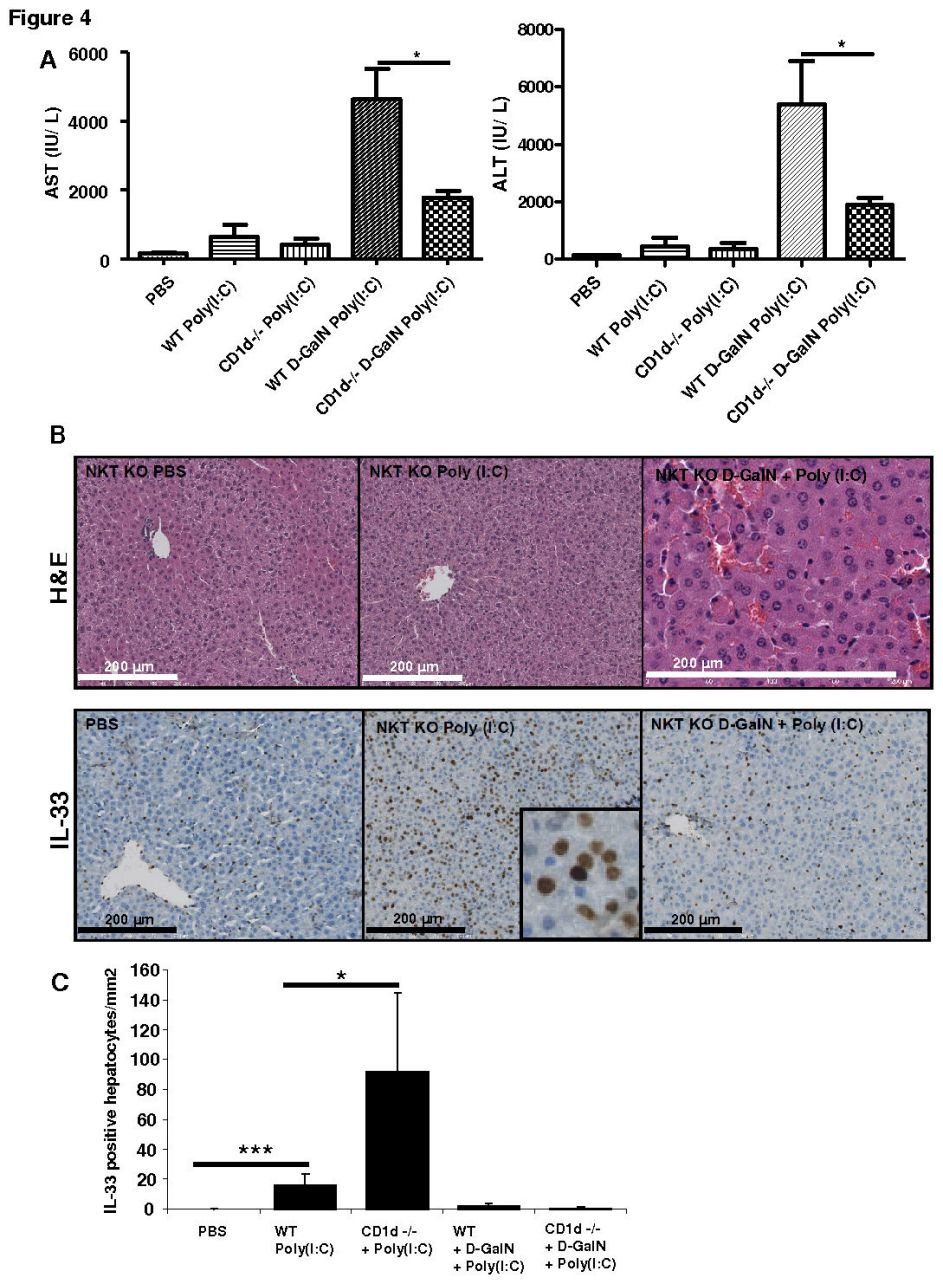
Liver injury and IL-33 expression in WT and NKT (CD1d) KO mice following Poly(I:C) and D-GalN Poly(I:C) treatment. (**A**) Levels of serum AST/ALT (IU/L) in WT and CD1d KO mice following PBS, Poly(I:C) (30 µg/mouse i.v.) and/or D-GalN Poly(I:C) treatment at 8h of post injection. (**B**) Liver histology (H and E) and immunostaining of IL-33 in livers of CD1d KO mice treated with PBS, Poly(I:C) and/or D-GalN Poly(I:C). (**C**) Comparison of number of IL-33 expressing hepatocytes in WT and CD1d KO mice following PBS, Poly(I:C) and/or D-GalN Poly(I:C) treatment at 8h of post injection.

The role of NKT cells in Poly(I:C)-induced liver injury is not well known but we have observed a partial decrease in NKT cells percentages in anti-ASGM1-treated mice (Figure 4A). We aimed to verify the impact of NKT cells deficiency in Poly(I:C)-induced liver injury and IL-33 expression in liver in CD1d KO (NKT KO) mice. WT control and NKT KO mice showed increased liver injury (2000 to 5000 AST/ALT levels) after D-GalN Poly(I:C) administration with a milder hepatoprotection in NKT KO mice (p <0.05) when compared with WT treated mice (Figure 4A). Liver histology revealed hemorrhagic lesions in liver after D-GalN Poly(I:C) treatment in NKT KO mice without remarkable liver injury in Poly(I:C) or vehicle control NKT KO mice (Figure 4B, upper panel). The hepatocyte-specific IL-33 expression was evident in Poly(I:C)-administered NKT KO mice but not in D-GalN Poly(I:C)-treated or control NKT KO mice (Figure 4B, lower panel). The number of IL-33 expressing hepatocytes significantly increased in Poly(I:C)-administered NKT KO mice compared to WT controls (p <0.05) but not in D-GalN Poly(I:C)-treated or control NKT KO mice (Figure 4C). These results suggested that NKT cells have a protective effect on liver injury in association with increased expression of IL-33 in hepatocytes during Poly(I:C)-induced liver injury.

#### L2-MHV3 induced fulminant hepatitis in mice was associated with increased expression of IL-33 in liver

**Figure 5:**
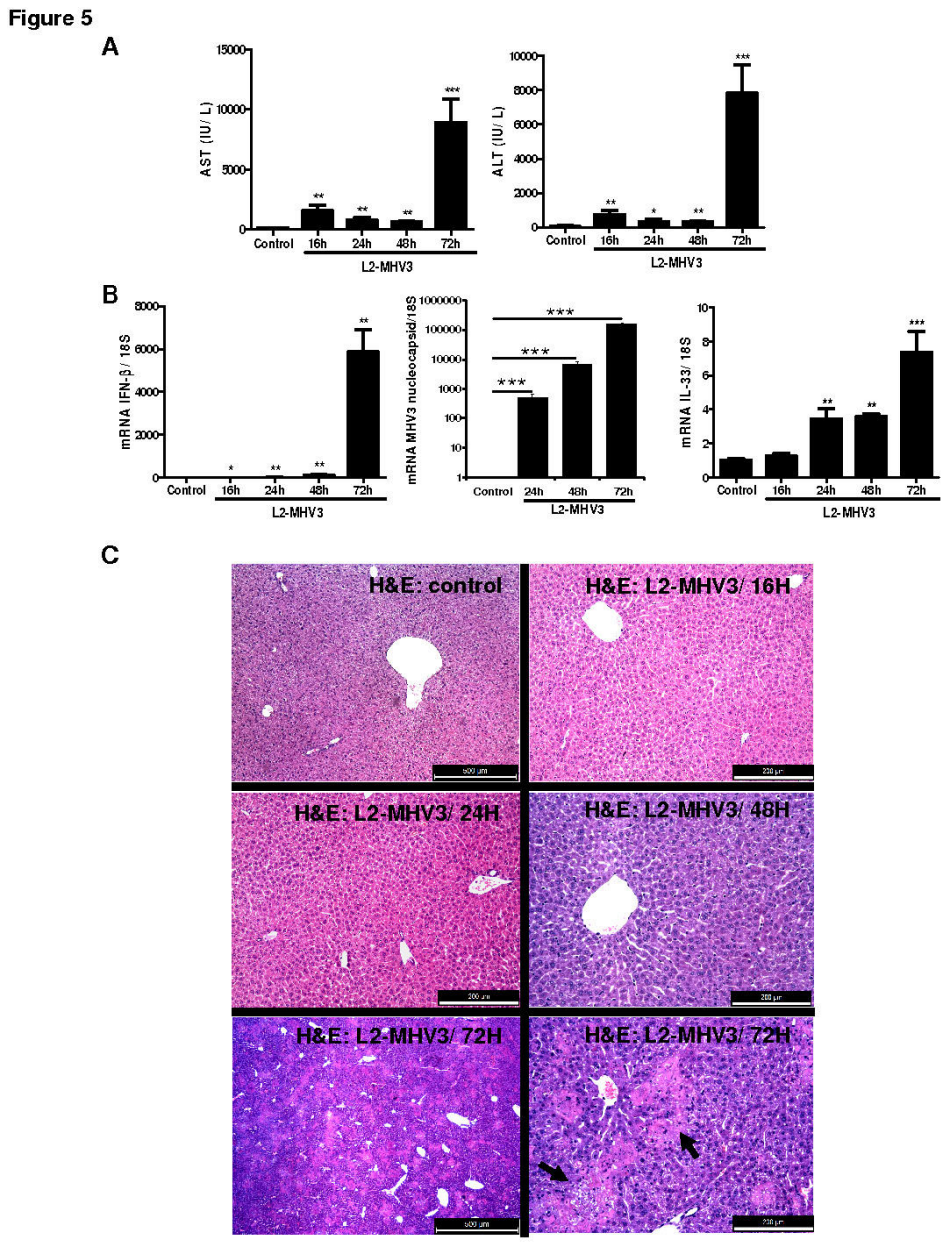
Liver injury and IL-33 expression in L2-MHV3 induced fulminant hepatitis in mice. (**A**) Levels of serum AST/ALT (IU/L) in mice infected with L2-MHV3 (10^3^ TCID(50)) or vehicle at 0, 16, 24, 48 and 72h of post infection. (**B**) Relative fold change in kinetics of mRNA expression of IFN-β, nucleocapsid of MHV3 and IL-33 in livers of L2-MHV3 induced hepatitis. (**C**) Sections of mice liver following vehicle or L2-MHV3 infection (16, 24, 48 and 72h) were stained with H&E for histopathology (arrows indicating zone of liver injury). (**D**) Immunolocalisation of IL-33 in livers of L2-MHV3 fulminant hepatic tissues by using primary antibody goat IgG anti-mouse-IL-33 and secondary HRP-conjugated rabbit anti-goat antibody with hematoxylin counterstaining (black arrows and red arrows indicating IL-33 positive hepatocytes and vascular/sinusoidal endothelial cells, respectively)). Scale bar was 50 µm. (**E**) Comparison of number of IL-33 expressing hepatocytes in vehicle and L2-MHV3 fulminant hepatic tissues (16, 24 and 32h).

The pathogenic strain of mouse hepatitis virus (L2-MHV3) induces fulminant hepatitis in C57BL/6 mice [23] and mimics a model of fulminant viral hepatitis (HBV) in human. We investigated the expression and cellular sources of IL-33 in L2-MHV3 induced acute hepatitis in C57BL/6 mice. The kinetics of L2-MHV3 infection in mice exhibited increase in serum AST/ALT levels following 16, 24, 48 and 72h of viral infection with severe and peak liver injury at 72h (p <0.001) (Figure 5A). Accordingly, the liver mRNA expressions of IFN-β and nucleocapsid of MHV3 that served as markers of viral infection, increased significantly at 16, 24, 48 and 72h of post infection with peak at 72h (Figure 5B). Interestingly, the mRNA expression of IL-33 was significantly up-regulated following L2-MHV3 infection reaching maximum at 72h post infection (p <0.001) (Figure 5B).

The histology of liver tissues showed important perivascular and parenchymal zone of liver injury at 72h of L2-MHV3 infection compared to vehicle control mice liver and no appreciable liver injury at 16, 24 and 48h time points (Figure 5C). The immunostaining of IL-33 in livers of L2-MHV3 infected mice revealed induced expression of IL-33 in liver sinusoidal endothelial cells, vascular endothelial cells and hepatocytes (Figure 5D). The liver sinusoidal endothelial cells and vascular endothelial cells represented inducible expression of IL-33 at 16, 24, 28, 32, 48 and 72h of L2-MHV3 infection compared to vehicle control mice (Figure 5D). However, the kinetics of hepatocyte-specific IL-33 expression was specifically found at 24, 28 and 32h of L2-MHV3 induced liver injury. In accordance, the number of IL-33 expressing hepatocytes following L2-MHV3 hepatitis increased significantly at 24, 28 and 32h of infection (Figure 5E). Our data showed that IL-33 expression is up-regulated in liver sinusoidal and vascular endothelial cells and hepatocytes during L2-MHV3-induced fulminant hepatitis.

### MHV3 infection in mice up-regulated pro-inflammatory cytokine expression in liver

**Figure 6:**
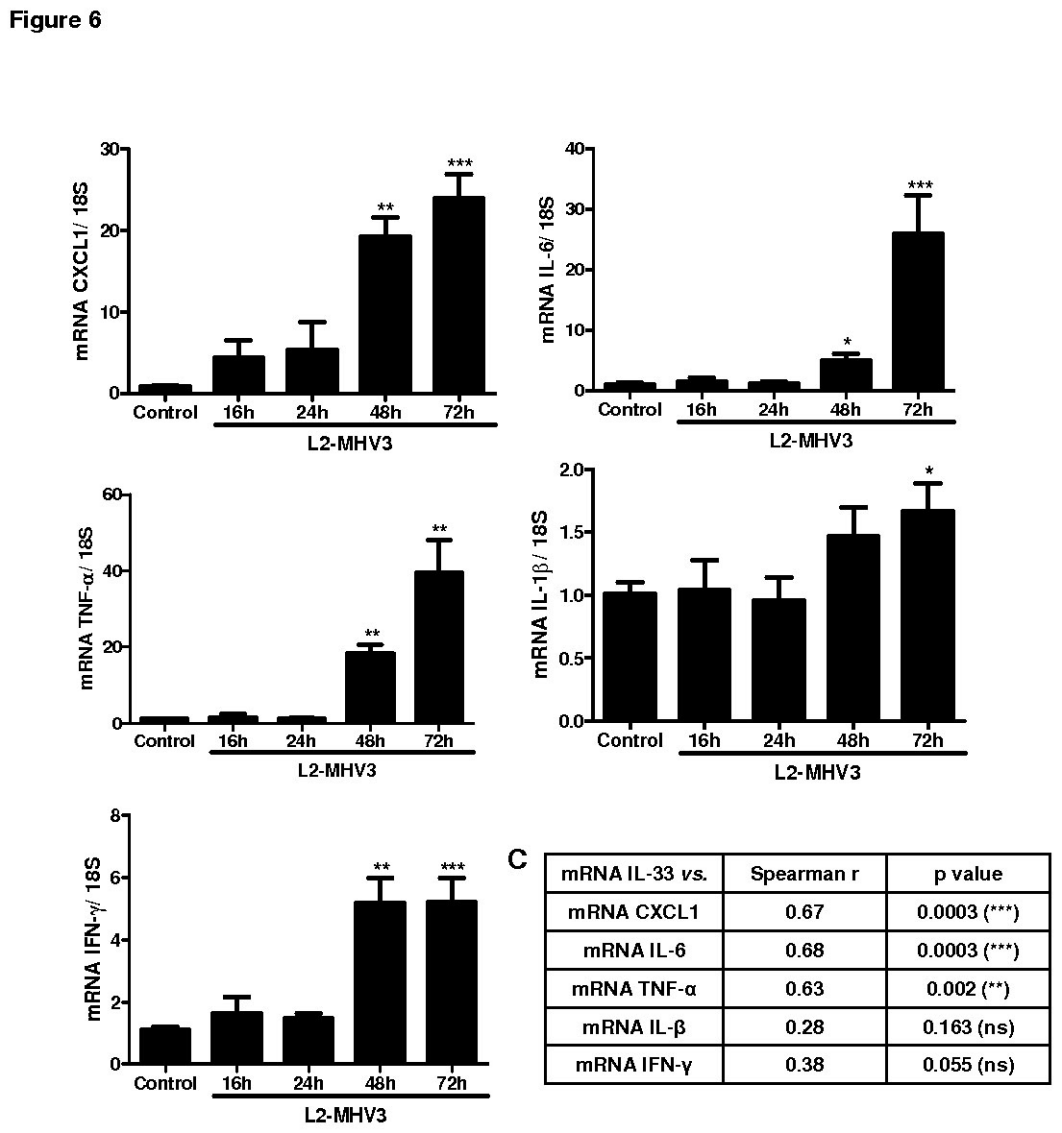
Cytokine expression of CXCL1, IL-6, TNF-α, IL-1β and IFN-γ in L2-MHV3 induced hepatitis mice. (**A**) Relative fold change in kinetics of mRNA expression of CXCL1, IL-6, TNF-α, IL-1β and IFN-γ in liver of L2-MHV3 infected mice at 0, 16, 24, 48 and 72h of post infection. The PBS-treated mice served as a reference for mRNA expression. (**B**) Spearman R correlation of mRNA expression of IL-33 with mRNA expression of CXCL1, IL-6, TNF-α, IL-1β and IFN-γ in liver of L2-MHV3 infected mice.

The inflammatory cytokines play an important role in development of fulminant hepatitis. Therefore, we studied the expression of pro-inflammatory cytokine expression in liver after L2-MHV3 infection. The kinetics of TNF-α, CXCL1, IFN-γ and IL-6 showed a similar time dependent increasing trend with peak expression at 72h of L2-MHV3 infection (Figure 6A). The mRNA expression of IL-1β was not greatly increased following L2-MHV3 hepatitis compared to control mice (Figure 6A). A significant correlation between mRNA expression of IL-33 and TNF-α, CXCL1 and IL-6 was evident but not with IL-1β or IFN-γ (Figure 6B). Hence, the elevated pro-inflammatory cytokine micro-environment is important for development of L2-MHV3-induced acute hepatitis in mice.

## Discussion

The over-expression of IL-33 and ST2 is associated with acute and chronic liver diseases in mice and human. IL-33 and sST2 have shown to be up-regulated in acute on chronic and chronic hepatic failure [15] and in chronic HBV and HCV infections in human [14,16,17]. The cellular sources of IL-33 in viral fulminant hepatitis are not well known. Accordingly, the murine fulminant hepatic models of TLR3 agonist, Poly(I:C), and pathogenic mouse hepatitis virus (L2-MHV3) are relevant acute viral hepatic models in human. Thus, we aimed to know the expression and regulation of IL-33 in Poly(I:C) and L2-MHV3 induced hepatitis in mice. The Poly(I:C) administration in mice induced moderate hepatic injury while co-administration of D-GalN and Poly(I:C) led to very severe fulminant hepatitis in mice. The liver injury induced by D-GalN Poly(I:C) treatment was associated with hemorrhagic lesions in liver and elevated pro-inflammatory cytokines as reported earlier [19,21,22]. The underlying mechanism of Poly(I:C)-induced liver injury is mediated by activation of Kuppfer cells and NK cells in a TLR3 dependent pathway [20] in association with increased inflammatory cytokines. Inducible expression of IL-33 was found in liver sinusoidal endothelial cells and vascular endothelial cells following Poly(I:C) and D-GalN Poly(I:C) treatment. However, hepatocyte-specific IL-33 expression was only evident in Poly(I:C) treated mice with increased number of IL-33 expressing hepatocytes compared to control mice. It may be plausible that hepatocyte specific inhibition of transcription by D-GalN prevented hepatocyte-specific IL-33 expression at transcript and protein level following D-GalN Poly(I:C) treatment. However, innate immune stimulation by the TLR3 agonist alone can induce IL-33 expression in liver especially in hepatocytes. The regulation of IL-33 by TLR viral and bacterial ligands have been demonstrated in human corneal epithelial cells and fibroblasts [30,31] as well as in murine macrophages [32]. The IL-33 was up-regulated by Poly(I:C) stimulation in murine macrophages and its transcriptional regulation was dependent of two transcription factors, IFN regulatory factor-3 (IRF-3) and CREB [32]. Here, we add TLR3 mediated expression of IL-33 in liver sinusoidal endothelial cells, vascular endothelial cells and hepatocytes in acute hepatitis in mice in a pathophysiological context. In addition, TLR3 expression is also rapidly increased in liver from L2-MHV3 infected mice (results not shown).

While in a viral murine model, IL-33 has been shown to be expressed by radio-resistant cells of the spleenic T cell zone in lymphocytic choriomeningitis virus (LCMV) infection [7], the above data prompted us to compare the IL-33 expression in a natural viral infection in liver in using the serotype 3 of mouse hepatitis viral infection MHV model [23,25]. We demonstrated the cellular expression of IL-33 in sinusoidal and vascular endothelial cells and hepatocytes at various times of the fulminant viral hepatitis induced by L2-MHV3. The L2-MHV3-induced liver injury was associated with significant increases in serum transaminases ALAT/ASAT, viral nucleocapsid, IFN-β and pro-inflammatory cytokine and chimiokine expression. The increased CXCL1 during MHV3 induced acute hepatitis may lead to chemotaxis/infiltration of neutrophils as an early response to liver infection and development of inflammatory microenvironment. The mechanism of MHV3-induced liver injury have shown to be dependent on activation of target cells of virus like Kupffer cells, NK cells, hepatocytes, sinusoidal endothelial and vascular endothelial cells [26,27]. Here we have shown that mRNA expression of IL-33 was over-expressed after L2-MHV3-induced hepatitis in mice. The transcript level of IL-33 was highly increased in L2-MHV3 induced hepatitis than Poly(I:C) treated mice demonstrating a difference between TLR-3 agonist and natural virus infection in liver. TLR3 expression is also rapidly increased in liver from L2-MHV3 infected mice (results not shown), suggesting that another factor may be also involved in the increase of IL-33. The hepatocyte-specific expression of IL-33 in hepatocytes was associated with beginning of L2-MHV3 induced liver injury (24, 28 and 32h) and the inducible expression of IL-33 in liver sinusoidal endothelial cells and vascular endothelial cells was sustained during the whole infection period (16 to 72h).

We next studied the role of NK and NKT cells in Poly(I:C) induced IL-33 expression in liver. The depletion of NK cells by anti-AGSM1 antibody in mice did not inhibit increased liver injury in D-GalN sensitized Poly(I:C) treated mice and had any effect in Poly(I:C) alone administration. These results are contrary to earlier findings which showed that pre-depletion of NK cells protected mice against D-GalN Poly(I:C) induced liver injury [22]. The difference seemed to be related with kinetics and dose of D-GalN Poly(I:C) used in these studies. The significant increase in CXCL1 expression in NK-depleted mice may explain the interplay of immune cells migration in liver i.e. depletion of one immune cell population relatively compensate the other immune cell population. The major sources of CXCL1 in liver are endothelial cells, Kupffer cells, hepatic stellate cells, hepatocytes and neutrophils and CXCL1 in association with neutrophils is important for development of liver injury [33,34]. The IL-33 expression was induced in liver sinusoidal endothelial cells and vascular endothelial cells. The down-regulation of hepatocyte-specific IL-33 expression in NK depleted mice in Poly(I:C) treated mice suggests a partial regulation of IL-33 by NK cells. Interestingly, IL-33-expressing hepatocytes decreased at the same time than NK cells decreased in liver following L2-MHV3 infection [25], supporting the hypothesis of a regulatory role of NK cells in IL-33 expression in hepatocytes.

In a other relevant murine ConA-induced fulminant hepatic model, we have demonstrated that IL-33 is highly induced in liver especially in hepatocytes and the regulation of hepatocyte-specific IL-33 is dependent of NKT cells and TRAIL [10,13]. In the TLR-3 agonist model used in this study, NKT cells did not control IL-33 expression in Poly(I:C)-induced liver injury because NKT (CD1d) KO mice exhibited increased number of IL-33 expressing hepatocytes than WT controls. These results may suggest a protective role of NKT cells in association with increased hepatocyte-specific IL-33 expression during TLR3-mediated acute hepatitis. We and others have demonstrated such a protective role of NKT cells and IL-33 during ConA-induced liver injury model (Arshad et al. 2012, Hepatol, Volarevic et al. 2012, J Hepatol) that seems plausible in Poly(I:C)-induced acute liver injury. It has been previously reported that NKT cells did not decrease in liver following L2-MHV3 infection in mice in contrast to that observed with NK cells [25]. However, TRAIL level expression increased in Poly(I:C) treated mice, as previously observed in ConA-induced hepatic model [13].

In conclusion, the cytokine IL-33 is rapidly up-regulated during Poly(I:C) and MHV3-induced fulminant hepatitis in mice, suggesting that IL-33 may act as an alarmin. The liver sinusoidal endothelial cells, vascular endothelial cells and hepatocytes are potential sources of IL-33 during viral fulminant hepatitis and NK cells partially regulate hepatocyte-specific IL-33 expression.

## Supporting Information

Figure S1 **Survival curve of mice following D-GaIN, Poly(I:C) and D-GaIN Poly(I:C) treatment.**
(TIF)
